# Intrathecal morphine administration reduces postoperative pain and peripheral endocannabinoid levels in total knee arthroplasty patients: a randomized clinical trial

**DOI:** 10.1186/s12871-018-0489-5

**Published:** 2018-02-27

**Authors:** Martin Kaczocha, Syed Azim, James Nicholson, Mario J. Rebecchi, Yong Lu, Tian Feng, Jamie L. Romeiser, Ruth Reinsel, Sabeen Rizwan, Shivam Shodhan, Nora D. Volkow, Helene Benveniste

**Affiliations:** 1 Department of Anesthesiology, Stony Brook University, Stony Brook, New York, USA; 2 Department of Orthopaedics, Stony Brook University, Stony Brook, New York, USA; 3 Department of Applied Mathematics and Statistics, Stony Brook University, Stony Brook, New York, USA; 4 National Institute on Alcohol Abuse and Alcoholism, Bethesda, MD USA; 5 Department of Anesthesiology, Yale University, New Haven, CT USA

**Keywords:** Endocannabinoid, Pain, Total knee arthroplasty, Anandamide, Morphine

## Abstract

**Background:**

The primary goal of this study was to determine whether administration of intrathecal morphine reduces postoperative pain. The secondary goal was to determine the effect of intrathecal morphine upon circulating levels of the weakly analgesic endocannabinoids, anandamide (AEA) and 2-arachidonoylglycerol (2-AG), and the related lipids palmitoylethanolamide (PEA) and oleoylethanolamide (OEA).

**Methods:**

Forty two total knee arthroplasty (TKA) patients were enrolled in a prospective, double-blinded, randomized study. The intervention consisted of intrathecal morphine (200 μg) or placebo administered at the time of the spinal anesthesia. Postoperative pain was measured during the first 4 h after surgery while serum levels of AEA, 2-AG, PEA, OEA, and cortisol were measured at baseline and 4 h after surgery.

**Results:**

Administration of intrathecal morphine reduced postoperative pain 4 h after TKA surgery compared to placebo (*p* = 0.005) and reduced postoperative systemic opioid consumption (*p* = 0.001). At baseline, intrathecal morphine led to a significant reduction in AEA, 2-AG, and OEA levels but did not affect PEA or cortisol levels. In patients administered intrathecal placebo, 2-AG levels were elevated 4 h after surgery; whereas patients receiving intrathecal morphine showed reductions in AEA, PEA, and OEA when compared to placebo. At 4 h after TKA surgery cortisol levels were significantly elevated in the placebo group and reduced in those receiving morphine.

**Conclusions:**

These results indicate that intrathecal morphine reduces postoperative pain in TKA patients. Furthermore, activation of central opioid receptors negatively modulates the endocannabinoid tone, suggesting that potent analgesics may reduce the stimulus for production of peripheral endocannabinoids. This study is the first to document the existence of rapid communication between the central opioid and peripheral endocannabinoid systems in humans.

**Trial registration:**

This trial was registered retrospectively. Trial registry: NCT02620631. Study to Examine Pain Relief With Supplemental Intrathecal Morphine in TKA Patients, NCT02620631, 12/03/2015.

## Background

The endocannabinoid system regulates nociception in rodents and humans both through central and peripheral mechanisms [1–3]. Endocannabinoids are implicated in stress-induced analgesia such as that triggered by mild electric foot shock [4], from strenuous exercise [5], and in modulating the intertwined pain and emotional responses [6]. The endocannabinoids anandamide (AEA) and 2-arachidonoyl glycerol (2-AG) are endogenous lipids that serve as agonists for cannabinoid receptors (CB1 and CB2) [7, 8]. Blockade of endocannabinoid inactivating enzymes (monoacylglycerol lipase cleaves 2-AG; and fatty acid amide hydrolase cleaves AEA) elevates tissue endocannabinoid levels and produces antinociceptive effects [2, 9]. The N-acylethanolamines (NAEs) palmitoylethanolamide (PEA) and oleoylethanolamide (OEA) are lipids structurally related to AEA but do not activate cannabinoid receptors; instead they serve as agonists at the nuclear peroxisome proliferator-activated receptor alpha [10, 11]. Activation of peroxisome proliferator-activated receptor alpha by PEA produces antinociceptive effects in preclinical models of pain [11, 12]. Recent reports indicate that tissue PEA levels are suppressed in animal models of pain [13, 14] and circulating PEA levels are decreased in irritable bowel syndrome patients experiencing abdominal pain compared to pain-free patients [15], suggesting an antinociceptive role of PEA in certain active pain states.

Interactions between the endocannabinoid and opioid systems are well documented [16–18]. Previous preclinical studies demonstrated synergistic analgesic effects between opioids and cannabinoids and furthermore that analgesic effects of opioids can be reversed by blockade of cannabinoid receptors [19–23]. Clinical data also suggest that the opioid and cannabinoid systems regulate placebo-induced analgesia in humans [24, 25]. Endocannabinoid levels are dynamically regulated under chronic pain states [26, 27] and augmentation of endocannabinoid levels has been suggested as a novel therapeutic strategy for analgesic development [28, 29].

Total knee arthroplasty (TKA) is a procedure associated with significant acute and chronic postoperative pain [30–32]. Although the regulation of nociception between the endocannabinoid and opioid systems has previously been described, the influence of opioid receptor activation upon endocannabinoid levels in humans has never been examined. In a rat model of morphine sensitization, acute morphine administration elevated brain AEA levels while concomitantly decreasing 2-AG [33]. Herein, we examined whether acute intrathecal morphine administration, which suppresses postoperative pain in TKA patients [34], alters circulating levels of endocannabinoids prior to and after TKA surgery. Because the endocannabinoid tone may be recruited to dampen pain [35–37], and because the analgesic effects between opioids and cannabinoids are synergistic, we hypothesized that administration of intrathecal morphine would reduce postoperative pain in patients undergoing TKA by at least 30% [38] and consequently suppress circulating endocannabinoid levels.

## Methods

### Trial design

This study was conducted as a single-center, prospective, double-blind, placebo controlled randomized study with a two arm parallel group design to validate the previously described analgesic effects of intrathecal morphine [34] in our TKA patient population (Trial registry: www.clinicaltrials.gov NCT02620631). In addition, we performed ancillary analyses of assays for serum AEA, 2-AG, PEA, OEA, and cortisol at baseline and 4 h after surgery. No changes were made to the original clinical study design.

### Ethics, consent and permissions

All the experiments conducted were approved by the Stony Brook University institutional review board (#200362) and were performed in accordance with the Declaration of Helsinki (1964) [39]. Written consent was obtained from each patient.

### Study participants

Forty two patients scheduled for an elective unilateral TKA under spinal anesthesia and a femoral nerve block were enrolled for this study from March 24, 2011 to February 6, 2014. The participants were prospectively selected from the orthopedic clinic of Dr. Nicholson at the Joint Replacement Center, Department of Orthopaedics, Stony Brook University Hospital who also performed all of the surgical procedures. Patients were included if they fulfilled the following criteria: Adults between 18 and 80 years of age; ASA class 1–3; able to give informed consent and able to understand English. Patients with documented rheumatoid arthritis, patients scheduled for bilateral TKA, and patients scheduled for a TKA revision were excluded from the current study. In addition, we excluded patients allergic to morphine, morbidly obese patients (BMI > 45), patients with chronic respiratory disease, obstructive sleep apnea, patients with chronic pain with opioid usage of > 100 mg morphine-equivalents daily, and patients with a history of drug abuse. Demographic data, medical history, medications and other therapies used for treatment of pain were collected after consent was obtained from each patient’s electronic medical record at Stony Brook University Hospital. Patients were asked to abstain from all medication use for seven days prior to TKA surgery.

### Randomization

A computer-generated blocked randomization method was used to allocate subjects to the placebo or intrathecal morphine intervention. The allocation sequence was generated by random number tables by the study coordinators, and the allocation concealed in sealed envelopes. Patients were randomized to receive either placebo or 0.2 mg of intrathecal morphine with their standard spinal anesthesia. A pharmacist who was not otherwise involved in the study prepared the study drug after being given the randomization envelope by the study coordinator. The physician received the study drug for the spinal procedure and was unaware of the treatment administered; as were the enrolled patients.

### Intervention: Intrathecal morphine or placebo

Immediately prior to the surgery, at the time of the spinal anesthesia, blood was collected from the fasting patients. Intravenous sedation with midazolam (up to 2 mg) was administered approximately 5–10 min prior to the initiation of regional anesthesia after routine vital signs monitors had been positioned. All intrathecal injections were performed by a single operator. Spinal block: In either sitting or lateral patient positon subarachnoid bupivacaine 0.5% (12-15 mg) containing preservative-free morphine (0.2 mg) or placebo (sterile, preservative-free 0.9% NaCl) was administered using a 25 g spinal needle at the L3/L4 or L4/L5 interspace.

### Pre- and intraoperative management

All patients received a COX-2 selective inhibitor (Celecoxib) and 10 mg of oxycontin immediately prior to surgery. Surgical anesthesia was achieved by spinal anesthesia in addition to a femoral nerve block and intraoperatively all patients were sedated with intravenous propofol. Patient controlled analgesia was initiated in the post-anesthesia care unit and was available to the patients during the entire postoperative period. For more details see previous work [40, 41].

### Primary and secondary outcomes

The primary end point of the prospective placebo-controlled intrathecal morphine study was the patient’s subjective assessment of average postoperative pain at rest 4 h after TKA surgery evaluated using the numerical rating scale. Secondary endpoints included changes in circulating levels of AEA, 2-AG, PEA, OEA, and cortisol at 4 h after surgery from pre-surgical baseline; in addition to postoperative consumption of systemic opioids via patient controlled analgesia.

### Pain measurement

Pain was measured using a numerical rating scale (NRS, pain scored 0–10) which is widely used in the perioperative setting including at our hospital. The patient’s primary nurse, who was blinded to the treatment administered, obtained pain scores over the first 4 h after surgery using the NRS. The average of the pain scores obtained from 2 to 4 h after surgery is reported here as average pain at 4 h. Importantly, all of the pain scores were obtained when the patients were at rest.

### Quantification of serum endocannabinoids

Serum endocannabinoid levels were quantified as previously described [40, 41].

### Serum cortisol levels

Serum cortisol levels were measured using a Human Cortisol ELISA kit (BioVendor, Asheville, NC) according to manufacturer’s instructions. Each sample was measured in duplicate. The optical density of each well was read on a Multiskan FC Microplate Photometer (Fisher Scientific, Pittsburgh, PA) at 450 nm. The intra-assay coefficient of variation was calculated to be 2.19%, the inter-assay coefficient of variation was calculated to be 5.78%.

### Statistical analysis

To determine the sample size required for our study based on our hypothesis that the addition of 0.2 mg intrathecal morphine would reduce postoperative acute pain in TKA patients compared to placebo we performed a two sample independent t-test assuming homogenous variances. According to our published data [42], the average pain score was 4.6 ± 2.0 for patients with standard-of-care treatment (Group 2). We hypothesized a predicted pain improvement difference of 30% (of the Group 2 mean) in Group 1, which will yield an effect size (ratio of the mean difference and its standard deviation) of 0.7. At the significance level of α = 0.05, we would need 44 subjects in each group to achieve a power of 90% for the independent samples t-test. Given that the pain score measurements were obtained within 4 h after the end of surgery, we anticipated and compensated for 5% attrition, and planned to recruit 94 subjects (analysis executed using SAS 9.4).

Due to slower than expected recruitment of patients the study was terminated by Helene Benveniste and Syed Azim, who were subsequently unblinded and an interim analysis of the data was performed midway through the study. For the primary outcome, the analyses were performed according to the group to which the patients were randomized to and all data were included in the analysis, which is the intention to treat population. For the secondary outcome analyses, serum samples were not available for all patients; however, no imputations were made to account for missing observations. The effects of intrathecal morphine upon postoperative pain and postoperative morphine use were analyzed using the Mann Whitney test. All collected samples were subjected to endocannabinoid and cortisol analyses. The data are presented as mean ± standard deviation. NRS pain scores between placebo and morphine groups were analyzed by t-test. Repeated measures ANOVA (Mixed Effects Model Repeated Measures) was used to compare whether the changes in endocannabinoid and cortisol levels over time were different between placebo and morphine groups by testing the Group by Time interaction term. Estimated mean difference in change over time between morphine and placebo groups (and its 95% confidence interval) for each endocannabinoid and cortisol are presented. We considered a *p*-value < 0.05 as statistically significant and all analyses were conducted with SAS 9.4 and XLSTAT (Addinsoft, version 18.07).

## Results

**Fig. 1:**
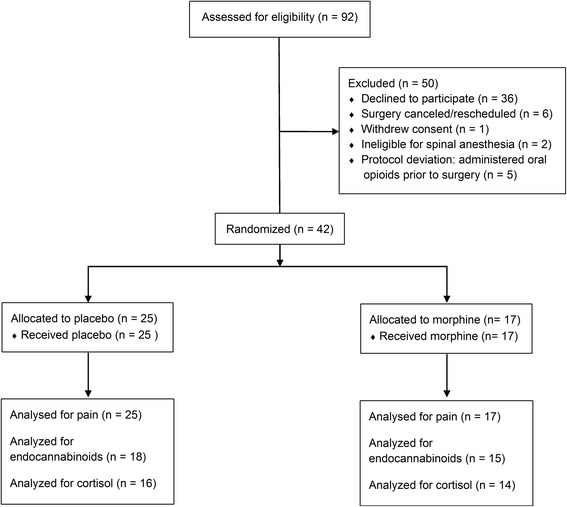
Consolidated Standards Of Reporting Trials flowchart of patient recruitment

**Fig. 2:**
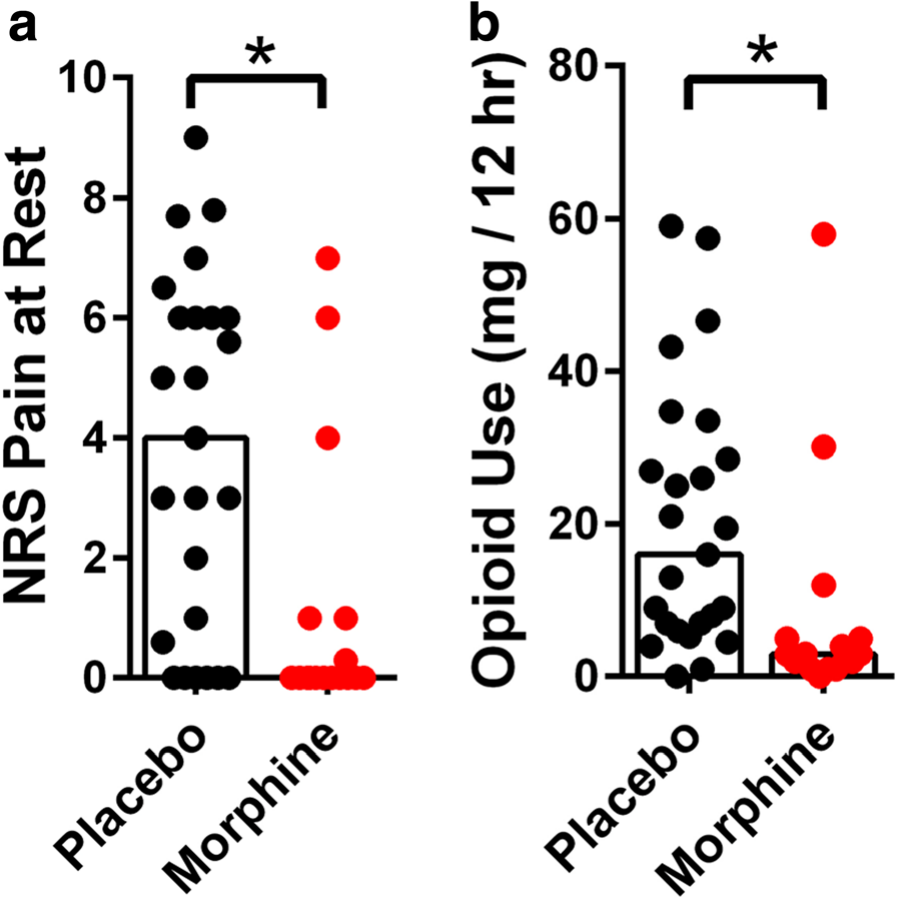
Postoperative pain at rest and analgesic use in patients receiving intrathecal morphine or placebo. **a** Average NRS pain scores were obtained during the first 4 h after TKA surgery. Bars represent the median and all data points are shown. Intrathecal morphine reduced acute postoperative pain (*p* = 0.0049). **b** Postoperative opioid use (morphine equivalent dose) measured via patient controlled analgesia during the first 12 h after surgery. Bars represent the median and all data points are shown. Intrathecal morphine reduced postoperative opioid use (*p* = 0.001)

**Fig. 3:**
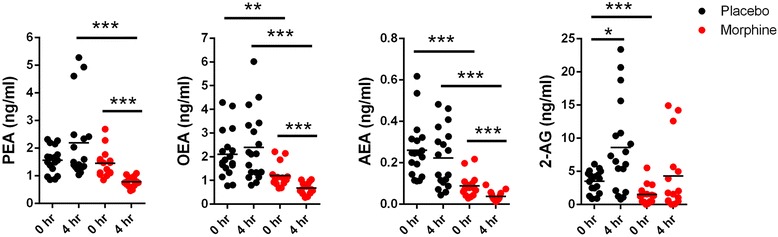
Serum levels of NAEs and endocannabinoids in patients receiving intrathecal morphine or placebo. The levels of PEA, OEA, AEA, and 2-AG were quantified in serum obtained preoperatively and 4 h after TKA surgery. *, *p* < 0.05; **, *p* < 0.01; ***, *p* < 0.001

Patients undergoing TKA were subdivided into two groups receiving either intrathecal morphine or placebo (Fig. 1). Table 1 shows key demographic features of the two patient groups. As expected and consistent with previous data [34], patients receiving intrathecal morphine reported reduced acute postoperative pain after surgery compared to placebo (Fig. 2a, *p* = 0.0049). Intrathecal morphine also reduced postoperative systemic opioid consumption measured using patient controlled analgesia (Fig. 2b, *p* = 0.001). Serum endocannabinoid and NAE levels were examined in patients receiving intrathecal morphine or placebo; and pre-surgical baseline (0 h) blood was drawn approximately 10–15 min after morphine administration. There were no differences in serum PEA levels between the placebo and morphine groups (Fig. 3, *p* = 0.532). In contrast, AEA (*p* < 0.0001), 2-AG (*p* = 0.0008), and OEA (*p* = 0.0025) levels were significantly lower in the morphine group at baseline compared to placebo (Fig. 3).

**Table 1:** Demographics of subjects selected for the study (Mean ± S.E)

	Placebo	Morphine
Number of Subjects	25	17
Gender (M/F)	6/19	8/9
Age	66.2 ± 1.8	64.0 ± 2.1
BMI (kg/m^2^)	32.2 ± 1.1	31.2 ± 1.3
History of Mood Disorders (Depression and/or Anxiety)	8	7
Preoperative Opioid Use	5	1

**Fig. 4:**
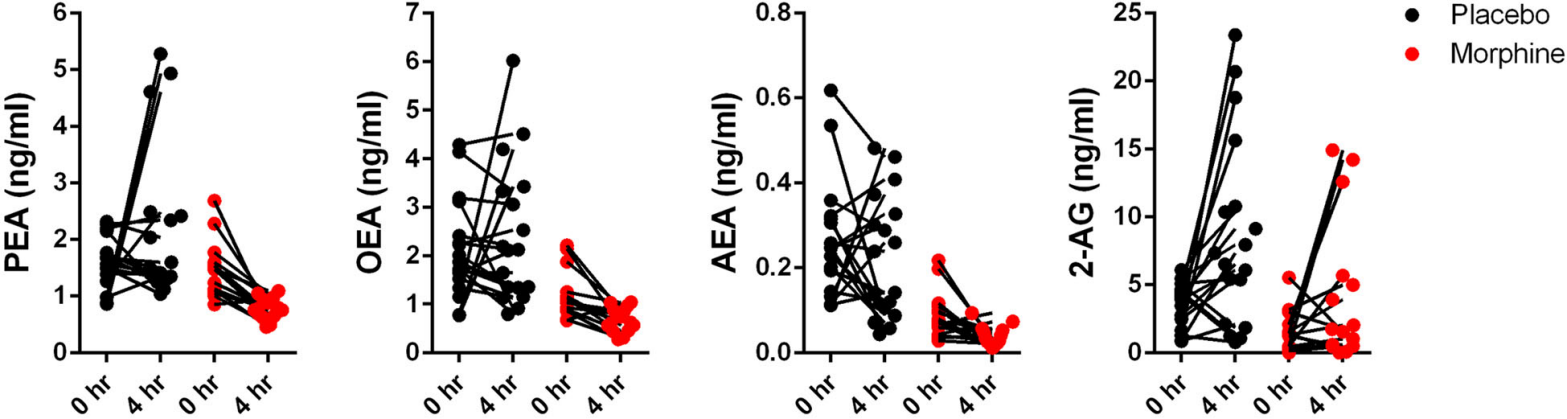
Trajectories of serum NAE and endocannabinoid levels. Levels of serum PEA, OEA, AEA, and 2-AG levels for each patient at baseline and 4 h after TKA surgery

We subsequently examined whether intrathecal morphine altered endocannabinoid levels 4 h after surgery. In the placebo group, 2-AG levels were significantly elevated (*p* = 0.008) 4 h after surgery while the change in levels of AEA (*p* = 0.424), PEA (*p* = 0.143), and OEA (*p* = 0.474) were not significant (Fig. 3 and Table 2). In contrast, compared to baseline, levels of AEA (*p* = 0.009), PEA (*p* = 0.0001), and OEA (*p* = 0.0008) were significantly reduced in the morphine group while the change in 2-AG levels was not significant (*p* = 0.0698). Trajectories of changes in serum endocannabinoid levels between the 0 and 4 h time points are shown in Fig. 4. Compared to placebo, the levels of AEA, PEA, and OEA were significantly lower in patients treated with intrathecal morphine at 4 h (Fig. 3). A significant group by time effect was observed for PEA (*p* = 0.0072) for the placebo and morphine groups (Table 2).

**Table 2:** Summary of serum PEA, OEA, AEA, 2-AG, and cortisol levels at baseline and 4 h after TKA surgery. Group by time interactions for each metabolite are also shown

	Time	Placebo			Morphine			Estimated Mean Difference in Change Over Time (95% Confidence Interval)	*P*-value (ANOVA Interaction)
		Mean	SD	N	Mean	SD	N		
PEA	Baseline	1.561	0.481	17	1.452	0.497	15	−1.310 (−2.237, −0.382)	0.0048
	4 h post-op	2.194	1.386	17	0.775	0.184	15		
OEA	Baseline	2.101	1.009	18	1.201	0.484	15	−0.819 (−1.712, 0.073)	0.0706
	4 h post-op	2.392	1.438	18	0.673	0.245	15		
AEA	Baseline	0.260	0.139	18	0.089	0.055	15	−0.014 (−0.116, 0.089)	0.7856
	4 h post-op	0.223	0.144	18	0.038	0.023	15		
2-AG	Baseline	3.502	1.605	18	1.499	1.465	15	−2.286 (−6.617, 2.145)	0.3008
	4 h post-op	8.568	6.932	18	4.279	5.282	15		
Cortisol	Baseline	13.250	7.176	16	14.025	5.528	14	−13.730 (−23.602, −3.859)	0.0081
	4 h post-op	22.937	13.712	16	9.981	6.650	14

**Fig. 5:**
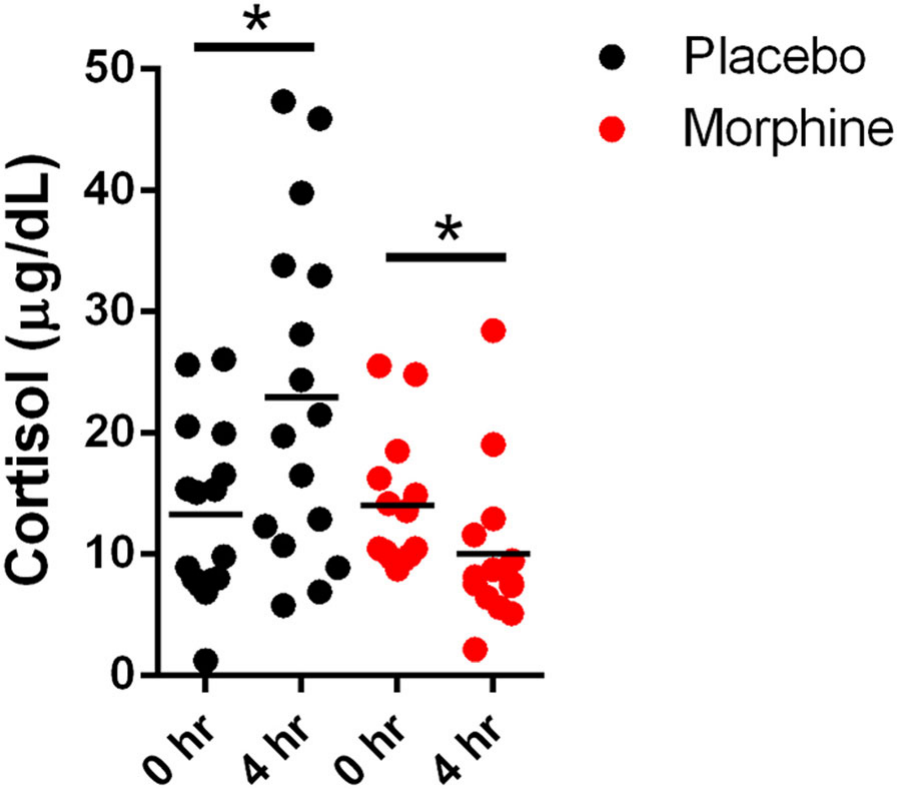
Serum cortisol levels in patients receiving intrathecal morphine or placebo. Levels of cortisol were quantified at baseline and 4 h after TKA surgery. *, *p* < 0.05

Surgical procedures produce a stress response that manifests as an elevation in circulating cortisol levels [43]; and corticosteroids enhance the biosynthesis of endocannabinoids [44–46]. Therefore, it is possible that the lower endocannabinoid levels observed in the morphine group may have resulted from a suppressed cortisol tone secondary to less acute pain. At baseline, there was no difference in cortisol levels (*p* = 0.7457) between the placebo and morphine groups (Fig. 5). However, cortisol levels were elevated 4 h after surgery in the placebo group (*p* = 0.043) while they were reduced in the morphine group (Fig. 5, *p* = 0.044). A significant group by time effect (*p* = 0.0103) was observed for cortisol for the placebo and morphine groups (Table 2); suggesting a reduced stress response in the patients receiving intrathecal morphine.

## Discussion

Our data demonstrate that intrathecal morphine reduced postoperative pain and the need for systemic opioids and altered baseline and postoperative levels of endocannabinoids in TKA patients. We demonstrated that intrathecal morphine lowered post-surgical levels of AEA, PEA, and OEA when compared to placebo. Interestingly, 2-AG as well as cortisol levels increased in TKA patients treated with placebo whereas 2-AG levels were not significantly altered and cortisol levels decreased in patients treated with morphine. These results are noteworthy given our previous data demonstrating that 2-AG is higher in CSF and synovial fluid in patients who report more severe postoperative pain after TKA surgery [41], possibly due to its conversion into downstream proalgesic eicosanoids [47].

Our novel data from human subjects agree with preclinical and clinical evidence consistently documenting crosstalk between the endocannabinoid and opioid systems. However, to date only a single preclinical study has examined the effects of opioids on endocannabinoid levels in rats [33]. To our knowledge, this is the first study to examine the effects of spinal opioid receptor activation upon circulating endocannabinoid/NAE levels. We hypothesized that morphine administration would reduce postoperative pain and bypass the need to recruit analgesic endocannabinoids.

The majority of patients scheduled for TKA surgery experience chronic pain due to end-stage osteoarthritis and endocannabinoid levels are known to be elevated in the blood and tissues of patients with chronic pain [35–37, 48]. In our study, patients that received intrathecal morphine had significantly lower levels of circulating endocannabinoids, which were measured approximately 10 to 15 min after morphine administration. Therefore, it is tempting to speculate that activation of central opioid receptors by morphine results in a rapid onset of analgesia that supplants the necessity to recruit endogenously produced analgesic endocannabinoids. Indeed, compared to patients receiving intrathecal placebo, the morphine group reported reduced postoperative pain 4 h after surgery that was accompanied by a suppressed endocannabinoid/NAE tone.

In our study, the lower preoperative levels of AEA, 2-AG, and OEA observed in subjects receiving intrathecal morphine are unlikely to stem from downregulation of endocannabinoid biosynthetic enzymes or upregulation of catabolic enzymes. Instead, the most plausible explanation is that morphine interferes with the activity of cells that produce endocannabinoids. A recent report found a similarly rapid reduction in AEA levels in the amygdala of mice after administration of corticotropin-releasing hormone [49]. The reduction in AEA levels was likewise rapid (within 10 min) and these effects were likewise independent of de novo synthesis of the endocannabinoid catabolizing enzyme fatty acid amide hydrolase.

Currently, the source(s) of systemically circulating endocannabinoids is not well defined. Previous work has demonstrated that leukocytes produce and secrete endocannabinoids [50, 51]. Intrathecal morphine has been shown to reduce leukocyte function in humans [52], which could theoretically account for the reduced endocannabinoid levels in our cohort. However, a high dose of morphine (0.5 mg) was required to establish this immunosuppressive effect, which likewise only manifested twenty four hours but not two hours after surgery [52]. Therefore, it is unlikely that leukocyte suppression accounts for the rapid reduction in circulating endocannabinoids after morphine administration.

Endocannabinoids are also produced in primary sensory neurons in a calcium-dependent manner [1, 53, 54]. Therefore, it is possible that activation of spinal opioid receptors reduced the excitability of primary sensory neurons [55] and consequently suppresses endocannabinoid/NAE production. Because endocannabinoids can regulate nociception at the level of the primary sensory neuron [1], the analgesic effects of morphine may dampen local biosynthesis of endocannabinoids.

General anesthesia reduces circulating AEA and augments 2-AG levels during the postoperative period [56–58]. In our patient cohort receiving intrathecal placebo, there were no changes in AEA, PEA, or OEA levels between the preoperative baseline and the 4 h postoperative time point while 2-AG levels as well as cortisol were elevated at 4 h. In contrast, the morphine group showed a significant suppression of AEA, PEA, and OEA levels, no 2-AG upregulation, and a decrease in cortisol postoperatively. These data suggest that the alterations in endocannabinoid levels are unlikely to stem from a general suppressive effect of anesthesia.

Surgical procedures induce a rapid increase in circulating cortisol levels and more effective analgesia blunts this postoperative cortisol response [43, 52, 59]. Interactions between stress and the endocannabinoid system are well established in rodents [60, 61]. Furthermore, acute stress in pain-free individuals stimulates cortisol release that is accompanied by a rapid increase in circulating AEA, OEA, and PEA levels [44, 46, 62]. Interestingly, a heightened cortisol response has been associated with lower pain in human subjects [63]. Therefore, in the morphine treated group, the endogenous postsurgical analgesic response that is normally characterized by release of cortisol and endocannabinoids, may have been supplanted by the potent analgesia attained after morphine administration.

One major limitation of the current study is the lack of baseline circulating endocannabinoid levels measured prior to intrathecal morphine or placebo administration. Another limitation stems from our inability to demonstrate that the suppression of the endocannabinoid tone after intrathecal morphine administration arises from its analgesic effects. Although we speculate that the reduction in endocannabinoids after morphine administration may reflect morphine analgesia and reduced necessity to recruit analgesic endocannabinoids, our study lacked patients without underlying chronic pain to validate this claim. We cannot rule out the possibility that the postsurgical increase in 2-AG in the placebo group might reflect pain-induced stress, which is known to increase 2-AG production [64] and which might have been blocked by intrathecal morphine as evidenced by the decrease in postsurgical cortisol levels in the patients who underwent the active treatment. Another limitation stems from the gender imbalance in favor of female subjects in the placebo group; therefore, the outcomes of this study may not completely extend to the male population and this must be controlled better in future more rigorous trials. Another limitation stems from the premature decision to stop the trial due to slower than expected recruitment; and incomplete dataset for the secondary outcome analyses of endocannabinoid and cortisol levels between the placebo and morphine groups. Lastly, the patients in the placebo group utilized more postoperative opioids to control their pain, which may have altered the levels of endocannabinoids. However, it is noteworthy that the baseline endocannabinoid levels in the placebo group are comparable to those observed in our previous study of TKA patients who were administered oral opioids prior to TKA surgery [41].

## Conclusions

In conclusion, our study demonstrates that intrathecal morphine significantly reduces acute postoperative pain after TKA surgery. In addition, this study is the first to document a previously undescribed regulation of peripheral endocannabinoid levels by spinal opioid receptor activation and extends the previously documented cannabinoid-opioid interactions observed in rodents and non-human primates to humans [16–18, 20, 21]. Our results indicate that activation of central opioid receptors negatively modulates the endocannabinoid tone, which may suggest that activation of strongly analgesic opioid receptors may obviate the need to recruit weakly analgesic endocannabinoids.

## Abbreviations

2-AG: 2-arachidonoylglycerol
AEA: Anandamide
CB1: Cannabinoid receptor 1
CB2: Cannabinoid receptor 2
OEA: Oleoylethanolamide
PEA: Palmitoylethanolamide
TKA: Total knee arthroplasty
